# *cis*-Bis(nitrato-κ^2^ *O*,*O*′)bis­(triethyl­phosphine oxide-κ*O*)nickel(II)

**DOI:** 10.1107/S1600536809015724

**Published:** 2009-05-07

**Authors:** Rüdiger W. Seidel

**Affiliations:** a Analytische Chemie, Ruhr-Universität Bochum, Universitätsstrasse 150, 44780 Bochum, Germany

## Abstract

In the title compound, [Ni(NO_3_)_2_(C_6_H_15_OP)_2_], the Ni^II^ ion, lying on a crystallographic twofold axis, adopts a distorted octa­hedral coordination, consisting of *O*-donor atoms of two symmetry-related triethyl­phospine oxide and two bidentate nitrate ligands.

## Related literature

For the synthesis and the crystal structure of the isotypic Co^II^ complex, see: Alnaji *et al.* (1991 ▶). For the preparation of the precursor *trans*-[NiCl_2_(Et_3_P)_2_] (Et_3_P = triethyl­phosphine), see: Jensen (1936 ▶). For the synthesis of *cis*-[Pt(NO_3_)_2_(Et_3_P)_2_], see: Kuehl *et al.* (2001 ▶).
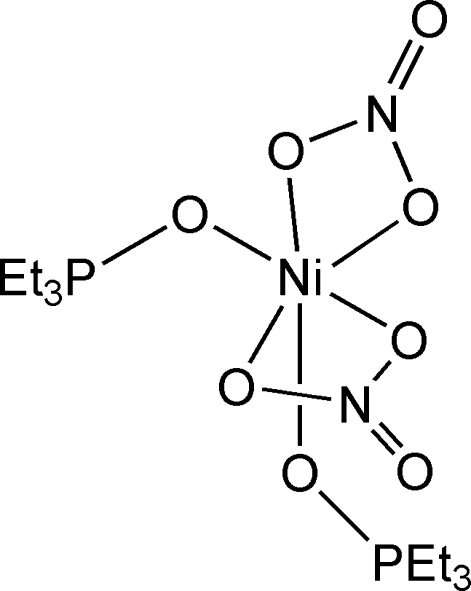


## Experimental

### 

#### Crystal data

[Ni(NO_3_)_2_(C_6_H_15_OP)_2_]
*M* *_r_* = 451.03
Monoclinic, 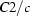

*a* = 16.954 (2) Å
*b* = 7.8494 (5) Å
*c* = 15.9905 (9) Å
β = 92.419 (5)°
*V* = 2126.1 (3) Å^3^
*Z* = 4
Mo *K*α radiation
μ = 1.10 mm^−1^
*T* = 294 K
0.31 × 0.26 × 0.24 mm

#### Data collection

Siemens P4 four-circle diffractometer
Absorption correction: ψ scan (*ABSPsiScan* in *PLATON*; Spek, 2009 ▶) *T*_min_ = 0.719, *T*_max_ = 0.770
2351 measured reflections
1829 independent reflections
1641 reflections with *I* > 2σ(*I*)
*R*_int_ = 0.047
3 standard reflections every 97 reflections intensity decay: none

#### Refinement

*R*[*F* ^2^ > 2σ(*F* ^2^)] = 0.029
*wR*(*F* ^2^) = 0.073
*S* = 1.05
1829 reflections
118 parameters
H-atom parameters constrained
Δρ_max_ = 0.22 e Å^−3^
Δρ_min_ = −0.28 e Å^−3^

### 

Data collection: *XSCANS* (Bruker, 1999 ▶); cell refinement: *XSCANS*; data reduction: *XSCANS*; program(s) used to solve structure: *SHELXS97* (Sheldrick, 2008 ▶); program(s) used to refine structure: *SHELXL97* (Sheldrick, 2008 ▶); molecular graphics: *DIAMOND* (Brandenburg, 2008 ▶); software used to prepare material for publication: *enCIFer* (Allen *et al.*, 2004 ▶).

## Supplementary Material

Crystal structure: contains datablocks I, global. DOI: 10.1107/S1600536809015724/kj2126sup1.cif

Structure factors: contains datablocks I. DOI: 10.1107/S1600536809015724/kj2126Isup2.hkl

Additional supplementary materials:  crystallographic information; 3D view; checkCIF report

## Figures and Tables

**Table:** 

Ni1—O1	1.9741 (16)
Ni1—O2	2.0738 (16)
Ni1—O3	2.1429 (17)

**Table:** 

O1—Ni1—O1^i^	94.2 (1)
O1—Ni1—O2^i^	96.97 (7)
O1—Ni1—O2	102.19 (6)
O2^i^—Ni1—O2	151.7 (1)
O1—Ni1—O3^i^	91.95 (7)
O2—Ni1—O3^i^	97.47 (7)
O1—Ni1—O3	162.74 (7)
O2—Ni1—O3	61.00 (7)
O3^i^—Ni1—O3	86.8 (1)

Symmetry code: (i) 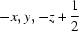.

**Fig. 1.**
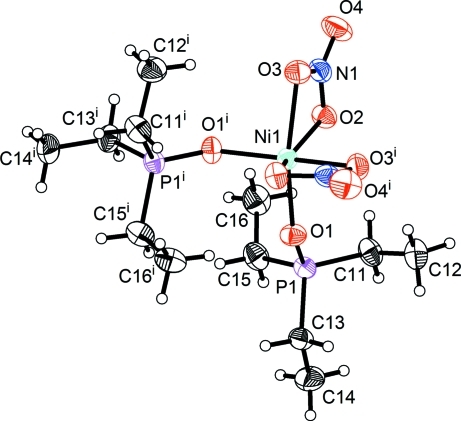
ORTEP diagram of the title compound. Displacement ellipsoids are drawn at the 30% probability level. Hydrogen atoms are drawn with arbitrary size. Symmetry codes: (i) -x,y,-z + 1/2.

## supplementary crystallographic information

### Comment

The title complex, *cis*-[Ni(NO_3_)_2_(OPEt_3_)] (OPEt_3_ = triethylphosphine oxide), exhibits *C*_2_ point symmetry and is located on a twofold crystallographic axis. An *ORTEP* diagram is depicted in Fig. 1.

As previously observed in the isotypic Co^II^ complex (Alnaji *et al.*, 1991), the triethylphosphine oxide and the nitrate ligands are arranged in a *cis* geometry about the Ni^II^ ion. The bond lengths are comparable to those reported for the Co^II^ complex. The bidentate nitrate ligand binds asymmetrically with Ni—O distances of 2.0738 (16) and 2.1429 (17) Å. This leads to differences in the N—O bond lengths. The N—O distances of the coordinated oxygen atoms are considerably longer than that of the free one (Table 1). The magnitude of the asymmetric bidentate binding is slightly smaller than that reported for the Co^II^ complex.

The O2—Ni1—O3 angle is small, as is expected for a bidentate chelating nitrate ion. The remaining angles about the Ni^II^ ion show large deviations from the regular octahedral geometry (see table 1). The P—O—Ni angle exhibits a typical value of 140.8 (1)°.

### Experimental

The title compound was obtained as an oxidation product of the metathesis reaction of *trans*-[NiCl_2_(Et_3_P)_2_] with AgNO_3_. The synthetic procedure was adapted from the preparation of *cis*-[Pt(NO_3_)_2_(Et_3_P)_2_] (Kuehl *et al.*, 2001). *trans*-[NiCl_2_(Et_3_P)_2_] was prepared according to the literature (Jensen, 1936). 33 mg (0.197) AgNO_3_ was added to a stirred solution of 36 mg (0.098 mmol) *trans*-[NiCl_2_(Et_3_P)_2_] in 40 ml acetone. The mixture was stirred 12 h in the dark. The colour changed from red to yellow. The solvent was removed in vacuum and the residue was suspended in 40 ml dichloromethane. Filtration and subsequent evaporation of the solvent yielded a yellow powder. A single-crystal suitable for X-ray diffraction was obtained from methanol-*d*4 when the solvent was allowed to evaporate slowly at ambient temperature.

### Refinement

Hydrogen atoms were placed at geometrically calculated positions and refined with *U*_iso_ 1.2 times (1.5 for methyl groups) of their parent atoms and allowing to ride on them. The initial torsion angles of the methyl groups were determined *via* a difference Fourier analysis and refined, while retaining the tetrahedral geometry.

### Crystal data

**Table:**

[Ni(NO_3_)_2_(C_6_H_15_OP)_2_]	*F*(000) = 952
*M**_r_* = 451.03	*D*_x_ = 1.409 Mg m^−^^3^
Monoclinic, *C*2/*c*	Mo *K*α radiation, λ = 0.71073 Å
Hall symbol: -C 2yc	Cell parameters from 50 reflections
*a* = 16.954 (2) Å	θ = 4.7–16.8°
*b* = 7.8494 (5) Å	µ = 1.10 mm^−^^1^
*c* = 15.9905 (9) Å	*T* = 294 K
β = 92.419 (5)°	Prism, yellow
*V* = 2126.1 (3) Å^3^	0.31 × 0.26 × 0.24 mm
*Z* = 4	

### Data collection

**Table:**

Siemens P4 four-circle diffractometer	1641 reflections with *I* > 2σ(*I*)
Radiation source: fine-focus sealed tube	*R*_int_ = 0.047
graphite	θ_max_ = 25.0°, θ_min_ = 2.9°
ω scans	*h* = −1→20
Absorption correction: ψ scan (ABSPsiScan in *PLATON*; Spek, 2009)	*k* = −1→9
*T*_min_ = 0.719, *T*_max_ = 0.770	*l* = −18→18
2351 measured reflections	3 standard reflections every 97 reflections
1829 independent reflections	intensity decay: none

### Refinement

**Table:**

Refinement on *F*^2^	Secondary atom site location: difference Fourier map
Least-squares matrix: full	Hydrogen site location: inferred from neighbouring sites
*R*[*F*^2^ > 2σ(*F*^2^)] = 0.029	H-atom parameters constrained
*wR*(*F*^2^) = 0.073	*w* = 1/[σ^2^(*F*_o_^2^) + (0.0309*P*)^2^ + 1.435*P*] where *P* = (*F*_o_^2^ + 2*F*_c_^2^)/3
*S* = 1.05	(Δ/σ)_max_ < 0.001
1829 reflections	Δρ_max_ = 0.22 e Å^−^^3^
118 parameters	Δρ_min_ = −0.28 e Å^−^^3^
0 restraints	Extinction correction: *SHELXL97* (Sheldrick, 2008), Fc^*^=kFc[1+0.001xFc^2^λ^3^/sin(2θ)]^-1/4^
Primary atom site location: structure-invariant direct methods	Extinction coefficient: 0.0039 (5)

### Special details

**Table:**

Geometry. All e.s.d.'s (except the e.s.d. in the dihedral angle between two l.s. planes) are estimated using the full covariance matrix. The cell e.s.d.'s are taken into account individually in the estimation of e.s.d.'s in distances, angles and torsion angles; correlations between e.s.d.'s in cell parameters are only used when they are defined by crystal symmetry. An approximate (isotropic) treatment of cell e.s.d.'s is used for estimating e.s.d.'s involving l.s. planes.
Refinement. Refinement of *F*^2^ against ALL reflections. The weighted *R*-factor *wR* and goodness of fit *S* are based on *F*^2^, conventional *R*-factors *R* are based on *F*, with *F* set to zero for negative *F*^2^. The threshold expression of *F*^2^ > σ(*F*^2^) is used only for calculating *R*-factors(gt) *etc*. and is not relevant to the choice of reflections for refinement. *R*-factors based on *F*^2^ are statistically about twice as large as those based on *F*, and *R*- factors based on ALL data will be even larger.

### Fractional atomic coordinates and isotropic or equivalent isotropic displacement parameters (Å^2^)

**Table:**

	*x*	*y*	*z*	*U*_iso_*/*U*_eq_	
Ni1	0.0000	0.58558 (5)	0.2500	0.04465 (16)	
P1	0.10851 (4)	0.30215 (8)	0.15778 (3)	0.04913 (18)	
O1	0.08156 (10)	0.4144 (2)	0.22694 (10)	0.0585 (4)	
O2	−0.04216 (10)	0.6501 (2)	0.13055 (10)	0.0596 (4)	
O3	−0.08635 (10)	0.7839 (2)	0.23545 (11)	0.0644 (5)	
O4	−0.12961 (13)	0.8501 (3)	0.10934 (14)	0.0895 (6)	
N1	−0.08784 (12)	0.7647 (3)	0.15682 (13)	0.0587 (5)	
C11	0.13399 (18)	0.4234 (3)	0.06864 (16)	0.0681 (7)	
H11A	0.1582	0.3481	0.0290	0.082*	
H11B	0.0860	0.4682	0.0418	0.082*	
C12	0.18933 (19)	0.5692 (4)	0.0880 (2)	0.0799 (8)	
H12A	0.1668	0.6427	0.1286	0.120*	
H12B	0.1977	0.6323	0.0377	0.120*	
H12C	0.2389	0.5256	0.1100	0.120*	
C13	0.19220 (15)	0.1817 (3)	0.19631 (17)	0.0642 (7)	
H13A	0.2336	0.2609	0.2137	0.077*	
H13B	0.1772	0.1198	0.2457	0.077*	
C14	0.22625 (19)	0.0551 (4)	0.1355 (2)	0.0910 (10)	
H14A	0.1884	−0.0334	0.1233	0.137*	
H14B	0.2736	0.0059	0.1602	0.137*	
H14C	0.2383	0.1127	0.0847	0.137*	
C15	0.03471 (17)	0.1488 (4)	0.12138 (18)	0.0722 (7)	
H15A	0.0588	0.0715	0.0826	0.087*	
H15B	0.0189	0.0820	0.1689	0.087*	
C16	−0.03782 (17)	0.2241 (4)	0.0791 (2)	0.0831 (9)	
H16A	−0.0620	0.3019	0.1166	0.125*	
H16B	−0.0744	0.1347	0.0641	0.125*	
H16C	−0.0235	0.2838	0.0296	0.125*	

### Atomic displacement parameters (Å^2^)

**Table:**

	*U*^11^	*U*^22^	*U*^33^	*U*^12^	*U*^13^	*U*^23^
Ni1	0.0491 (2)	0.0437 (2)	0.0416 (2)	0.000	0.00704 (16)	0.000
P1	0.0584 (4)	0.0457 (3)	0.0434 (3)	0.0050 (3)	0.0032 (2)	0.0001 (2)
O1	0.0626 (9)	0.0619 (10)	0.0511 (9)	0.0137 (8)	0.0046 (7)	−0.0072 (8)
O2	0.0711 (11)	0.0575 (10)	0.0502 (9)	0.0081 (9)	0.0033 (8)	0.0022 (8)
O3	0.0675 (11)	0.0579 (10)	0.0683 (11)	0.0082 (8)	0.0083 (9)	−0.0071 (9)
O4	0.0899 (14)	0.0704 (12)	0.1056 (16)	0.0125 (11)	−0.0261 (12)	0.0235 (12)
N1	0.0599 (12)	0.0473 (11)	0.0682 (14)	−0.0036 (10)	−0.0041 (10)	0.0072 (10)
C11	0.096 (2)	0.0589 (15)	0.0500 (13)	−0.0035 (14)	0.0058 (13)	0.0047 (12)
C12	0.087 (2)	0.0734 (19)	0.0802 (19)	−0.0169 (16)	0.0137 (16)	0.0077 (15)
C13	0.0660 (16)	0.0587 (15)	0.0683 (16)	0.0138 (13)	0.0091 (12)	0.0080 (12)
C14	0.082 (2)	0.0632 (18)	0.129 (3)	0.0169 (15)	0.025 (2)	−0.0113 (18)
C15	0.0865 (19)	0.0573 (15)	0.0721 (17)	−0.0088 (14)	−0.0049 (14)	0.0011 (14)
C16	0.086 (2)	0.0774 (19)	0.084 (2)	−0.0173 (17)	−0.0143 (16)	−0.0025 (16)

### Geometric parameters (Å, °)

**Table:**

Ni1—O1	1.9741 (16)	C12—H12A	0.9600
Ni1—O1^i^	1.9741 (16)	C12—H12B	0.9600
Ni1—O2^i^	2.0738 (16)	C12—H12C	0.9600
Ni1—O2	2.0738 (16)	C13—C14	1.521 (4)
Ni1—O3^i^	2.1429 (17)	C13—H13A	0.9700
Ni1—O3	2.1429 (17)	C13—H13B	0.9700
P1—O1	1.5001 (16)	C14—H14A	0.9600
P1—C11	1.782 (2)	C14—H14B	0.9600
P1—C13	1.793 (2)	C14—H14C	0.9600
P1—C15	1.814 (3)	C15—C16	1.499 (4)
O2—N1	1.270 (3)	C15—H15A	0.9700
O3—N1	1.266 (3)	C15—H15B	0.9700
O4—N1	1.217 (3)	C16—H16A	0.9600
C11—C12	1.503 (4)	C16—H16B	0.9600
C11—H11A	0.9700	C16—H16C	0.9600
C11—H11B	0.9700		
			
O1—Ni1—O1^i^	94.2 (1)	H11A—C11—H11B	107.6
O1—Ni1—O2^i^	96.97 (7)	C11—C12—H12A	109.5
O1^i^—Ni1—O2^i^	102.19 (6)	C11—C12—H12B	109.5
O1—Ni1—O2	102.19 (6)	H12A—C12—H12B	109.5
O1^i^—Ni1—O2	96.97 (7)	C11—C12—H12C	109.5
O2^i^—Ni1—O2	151.7 (1)	H12A—C12—H12C	109.5
O1—Ni1—O3^i^	91.95 (7)	H12B—C12—H12C	109.5
O1^i^—Ni1—O3^i^	162.74 (7)	C14—C13—P1	116.2 (2)
O2^i^—Ni1—O3^i^	61.00 (7)	C14—C13—H13A	108.2
O2—Ni1—O3^i^	97.47 (7)	P1—C13—H13A	108.2
O1—Ni1—O3	162.74 (7)	C14—C13—H13B	108.2
O1^i^—Ni1—O3	91.95 (7)	P1—C13—H13B	108.2
O2^i^—Ni1—O3	97.47 (7)	H13A—C13—H13B	107.4
O2—Ni1—O3	61.00 (7)	C13—C14—H14A	109.5
O3^i^—Ni1—O3	86.8 (1)	C13—C14—H14B	109.5
O1—P1—C11	111.57 (12)	H14A—C14—H14B	109.5
O1—P1—C13	108.53 (11)	C13—C14—H14C	109.5
C11—P1—C13	109.97 (13)	H14A—C14—H14C	109.5
O1—P1—C15	113.50 (12)	H14B—C14—H14C	109.5
C11—P1—C15	106.55 (13)	C16—C15—P1	115.1 (2)
C13—P1—C15	106.58 (13)	C16—C15—H15A	108.5
P1—O1—Ni1	140.8 (1)	P1—C15—H15A	108.5
N1—O2—Ni1	93.42 (13)	C16—C15—H15B	108.5
N1—O3—Ni1	90.36 (13)	P1—C15—H15B	108.5
O4—N1—O3	122.7 (2)	H15A—C15—H15B	107.5
O4—N1—O2	122.1 (2)	C15—C16—H16A	109.5
O3—N1—O2	115.21 (19)	C15—C16—H16B	109.5
C12—C11—P1	114.3 (2)	H16A—C16—H16B	109.5
C12—C11—H11A	108.7	C15—C16—H16C	109.5
P1—C11—H11A	108.7	H16A—C16—H16C	109.5
C12—C11—H11B	108.7	H16B—C16—H16C	109.5
P1—C11—H11B	108.7		

Symmetry codes: (i) −*x*, *y*, −*z*+1/2.
